# From antidepressants and psychotherapy to oxytocin, vagus nerve stimulation, ketamine and psychedelics: how established and novel treatments can improve social functioning in major depression

**DOI:** 10.3389/fpsyt.2024.1372650

**Published:** 2024-10-14

**Authors:** Aleksandra Kupferberg, Gregor Hasler

**Affiliations:** ^1^ Molecular Psychiatry Lab, Faculty of Science and Medicine, University of Freiburg, Villars-sur-Glâne, Switzerland; ^2^ University Psychiatry Research Unit, Freiburg Mental Health Network, Villars-sur-Glâne, Switzerland; ^3^ Department of Neuropsychology, Lake Lucerne Institute, Vitznau, Switzerland

**Keywords:** major depressive disorder, impairments, psychedelics, vagus, ketamine, antidepressants, treatment

## Abstract

Social cognitive deficits and social behavior impairments are common in major depressive disorder (MDD) and affect the quality of life and recovery of patients. This review summarizes the impact of standard and novel treatments on social functioning in MDD and highlights the potential of combining different approaches to enhance their effectiveness. Standard treatments, such as antidepressants, psychotherapies, and brain stimulation, have shown mixed results in improving social functioning, with some limitations and side effects. Newer treatments, such as intranasal oxytocin, mindfulness-based cognitive therapy, and psychedelic-assisted psychotherapy, have demonstrated positive effects on social cognition and behavior by modulating self-referential processing, empathy, and emotion regulation and through enhancement of neuroplasticity. Animal models have provided insights into the neurobiological mechanisms underlying these treatments, such as the role of neuroplasticity. Future research should explore the synergistic effects of combining different treatments and investigate the long-term outcomes and individual differences in response to these promising interventions.

## 1 Introduction

Major depressive disorder (MDD) is a prevalent and disabling psychiatric condition that impairs the social functioning and well-being of millions of people worldwide. In addition to the core symptoms of depressed mood, anhedonia, and altered appetite and sleep, MDD is associated with social cognitive deficits and behavioral impairments that exacerbate the distress and hinder the recovery of affected individuals (1, 2). These social deficits are pervasive and encompass almost every aspect of one’s social capabilities, such as emotion regulation, social cognition, and social behavior. These impairments are more subtle than those observed in other neuropsychiatric disorders, such as autism and schizophrenia, but they may affect a larger proportion of patients and have a greater impact on their quality of life (3–6). For instance, MDD patients show similar attentional biases and difficulties in disengaging from negative emotional stimuli as autistic patients (7). MDD patients are also more vulnerable to interpersonal stressors, such as loss, humiliation, or rejection (8), which can undermine their self-worth and exacerbate their depressive symptoms (9). Additionally, interpersonal stressors have been found to be stronger predictors of future depression than non-interpersonal stressors, such as financial, academic, or health problems (10–12).

Impairments in various domains of social functioning, such as social support, network size, occupational and marital functioning persist even after remission from mood symptoms (13, 14). These residual symptoms not only reduce the quality of life of MDD patients but also increase the risk of relapse and chronicity (15). Social cognition deficits in MDD can emerge early in life and persist across development. Studies have shown that children and adolescents with high levels of depressive symptoms have more conflictual, negative, and less collaborative and mutual social interactions with peers than those with low levels of depressive symptoms (16–19). Thus, social impairment pose significant challenges to successful interpersonal functioning and quality of life for both depressed patients and their families (20, 21) and successful remission from MDD requires not only a decrease in depressive symptoms but also significant improvements in the social domain (22).

Conventional treatments for MDD, such as antidepressants, and some brain stimulation therapies, such as electroconvulsive therapy, have primarily focused on reducing mood symptoms and have shown variable and often limited effects on social functioning (23), with various drawbacks and side effects, including sexual dysfunction (24) or cardiovascular problems (25). In contrast, novel treatments, such as intranasal oxytocin, vagus nerve stimulation (VNS) and mindfulness-based cognitive therapy (MBCT), ketamine and psychedelic-assisted psychotherapy (PAP), have emerged as promising alternatives for enhancing social cognition and behavior in MDD (26–29). These innovative approaches target key aspects of social functioning, such as social bonding, emotion recognition, rumination, emotional regulation, empathy, and connectedness, with rapid and lasting effects. Animal models have complemented human studies by revealing the neurobiological mechanisms underlying the social effects of these treatments (30–32). Therefore, it is crucial to evaluate the effects of both conventional and novel therapeutic interventions on social outcomes in MDD.

In this article, we review the current literature on the impact of standard and novel treatments on social functioning in major depression. We first provide an overview of the social deficits associated with major depression and how they affect the patients, their families, and their friends. We then discuss the effects of conventional treatments, such as antidepressants, psychotherapies, and some brain electro-stimulation therapies, on social functioning. We highlight the limitations and challenges of these treatments and point out the need for alternative approaches. Next, we introduce novel treatments that have emerged as promising options for enhancing social functioning in major depression. These include intranasal oxytocin, vagus nerve stimulation, mindfulness-based cognitive therapy (MBCT), ketamine and psychedelic-assisted psychotherapy (PAP). We describe how these treatments target key aspects of social functioning, such as social bonding, emotion recognition, emotional regulation, empathy, and connectedness. We also present the evidence from animal models that reveal the neurobiological mechanisms underlying the social effects of these treatments. Finally, we identify the gaps and challenges for future research.

## 2 Methods

In this narrative review, we delve into two critical domains of social dysfunction observed in patients with Major Depressive Disorder (MDD): social cognition and social behavior. These domains play a pivotal role in shaping an individual’s ability to navigate social interactions and maintain healthy relationships. Dysfunction within these domains can significantly impair individuals’ social functioning. Specifically, social cognition encompasses a range of conscious and subconscious psychological processes that influence social behavior, including socio-emotional regulation strategies such as cognitive reappraisal, forgiveness capacity, decentering, empathy, sensitivity to social rejection, cognitive flexibility, emotion recognition deficits, and diminished self-compassion, ultimately impacting social connectedness.

We conducted a targeted literature search specifically focused on depression, deliberately excluding other mental disorders to maintain a narrower scope and reduce the length of the article. This approach allowed us to concentrate on the most relevant studies pertaining to depressive symptoms and treatments without the additional complexity of related conditions.

The search was conducted using several electronic databases, including PubMed and Web of Science. We used a precise set of search terms to locate relevant studies, including ‘depression,’ ‘major depressive disorder,’ ‘depressive symptoms,’ and ‘treatment.’ The inclusion criteria required that studies explicitly address depressive symptoms or treatments. This focused strategy was designed to ensure that our review remained concentrated on depression alone, providing a comprehensive yet specific overview of the literature.

In our review, we have used the term “novel” to refer to treatments that represent a significant departure from traditional therapies commonly used in clinical practice. This includes treatments that are either in the experimental stages or have only recently been introduced into clinical settings. However, our use of “novel” is not strictly limited to the recency of development but also encompasses the extent to which these treatments are integrated into routine clinical practice across different countries. Thus, the perception of novelty may vary based on individual perspectives and regional practices. For example, a treatment method that is considered well-established and widely used in one country may be relatively new or less commonly utilized in another country due to differences in healthcare policies, availability of resources, and cultural attitudes toward mental health treatment. Additionally, regulatory agencies in different countries may have varying standards for approving and endorsing treatment methods, which can influence their adoption and acceptance within the healthcare system. Thus, despite initial testing in research studies or clinical trials, some methods might not yet be widely adopted in clinical practice depending on the country. Novel methods may or may not be recent; they could have been developed recently or may have been around for some time but are only now gaining recognition or undergoing rigorous evaluation.

## 3 Impairments of social cognition in MDD

### 3.1 Decreased socio-cognitive reappraisal

**Figure 1:**
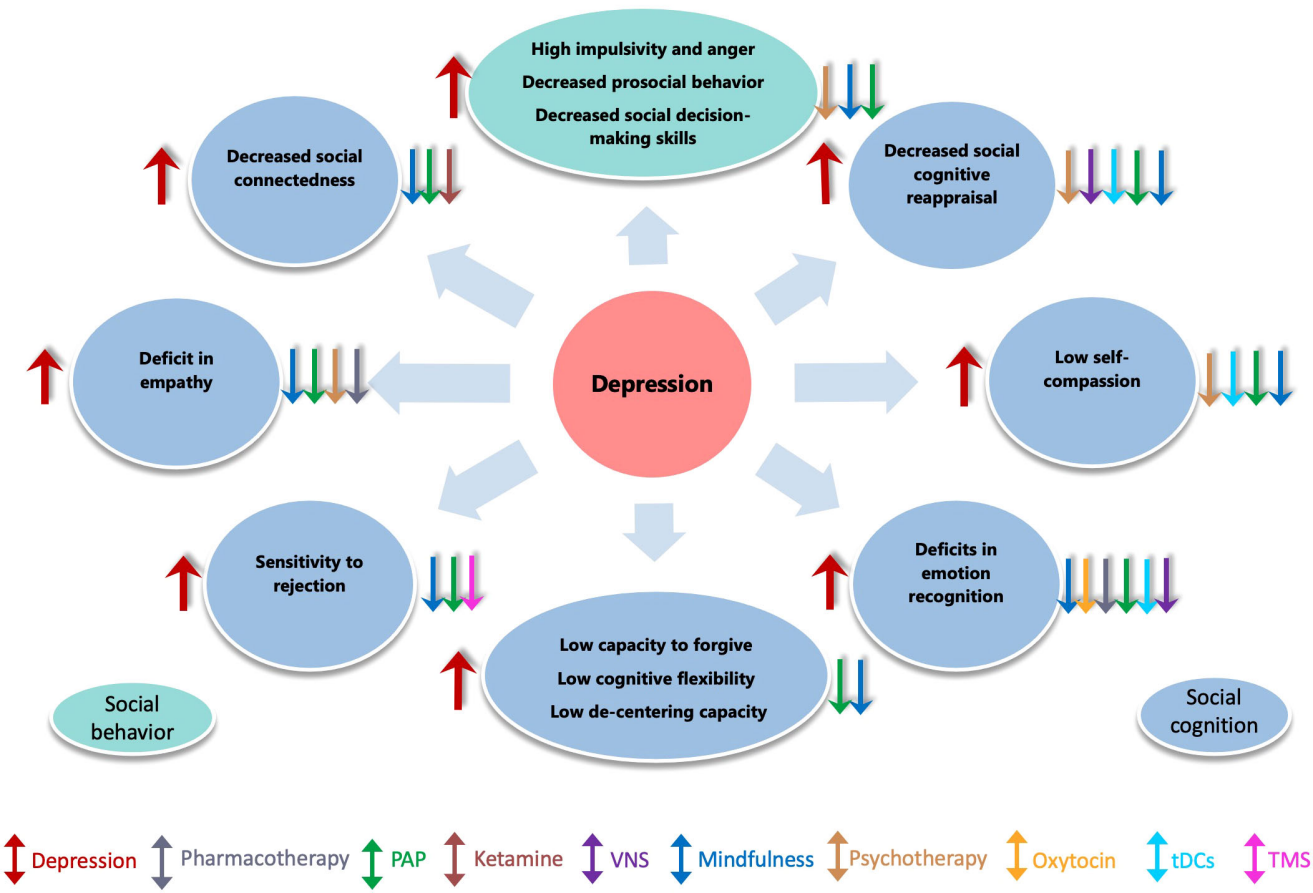
Impact of depression on social behavior, cognition, and the effect of treatment interventions. This figure illustrates how depression impairs empathy, emotion recognition, social connectedness,cognitive flexibility etc. (red arrows). Various interventions, including pharmacotherapy, psychotherapy, mindfulness, ketamine, psychedelic, brain stimulation techniques (TMS, tDCS, VNS) etc., are shown to help alleviate these effects.

The regulation of emotions is essential not only for momentary emotional experiences and behaviors but also for broader and more enduring aspects of psychological functioning (33, 34). Social cognitive reappraisal, a form of emotion regulation, involves changing one’s thoughts about a potentially distressing situation to minimize its emotional impact (35). This type of reappraisal is aimed at reinterpreting negative emotions, thoughts, or situations in a positive light (36). Studies have demonstrated that cognitive reappraisal attenuates negative emotions and promotes mental health (37). It has also been suggested that social difficulties in depression might arise from an ineffective use of socio-cognitive reappraisal (34, 38–40). Moreover, a lower inclination to use positive reappraisal has been consistently linked to subclinical and clinical forms of depression (37) (see **Figure 1**). Further, children’s use of cognitive reappraisal may also influence their risk of developing depression, with those who have never experienced a depressive episode being 1.3 times more likely to use cognitive reappraisal than those currently in a depressive episode (41). Therefore, encouraging the use of cognitive reappraisal may be a promising strategy for managing and preventing depression.

These findings suggest that cognitive reappraisal is important for mental health and social functioning because it can minimize emotional impact of social stressors. Therefore, it is important to address the deficits in cognitive reappraisal in the treatment of depression and to improve the social outcomes of depressed individuals.

### 3.2 Low decentering capability

Decentering is the ability to assume a detached, objective stance toward present-moment awareness, which allows individuals to experience distressing inner events as imperfect models of the real world rather than precise reflections (42). Repeated cognitive reappraisal has been shown to facilitate decentering, attenuate negative emotions and help individuals understand that emotions and thoughts are only temporary mental events that do not necessarily reflect one’s self or external reality (43, 44). Decentering allows individuals to experience distressing inner events as imperfect models of the real-world rather than precise reflections (45). Reduced decentering ability has been linked with several psychiatric disorders, including major depression (46, 47) (see **Figure 1**).

Individuals with a tendency toward self-rumination have difficulty spontaneously taking a decentered perspective and are consequently prone to depression (44, 48). Thus, decentering is of particular interest in depression since it may reduce levels of depressive rumination by teaching patients more adaptive ways of relating to their thinking (49). Additionally, decentering contributes to preventing the recurrence of depression (42). Notably, the ability to decenter is related to diminishing egocentrism, a state similar to ego-dissolution achieved by the use of psychedelics.

### 3.3 Low self-compassion and self-acceptance and high self-criticism

Depression is often characterized by a self-critical attitude, which can lead to emotional difficulties such as uncontrolled anger (50) (see **Figure 1**). Those who are caught up in self-evaluation may also be needy and devote considerable attention to self-aggrandizement in order to compensate for perceived personal deficits. Self-compassion, in contrast to self-criticism, is characterized by a non-judgmental attitude toward one’s inadequacies and failures, feelings of caring and kindness toward oneself, and recognition that one’s experience is part of the common human experience. Higher levels of self-compassion were shown to be related to lower levels of depressive symptoms (51). Thus, individuals with depression, both current and remitted, have significantly lower levels of self-compassion compared to those without depression. For example, higher levels of self-compassion have been associated with lower levels of depressive symptoms (52). Conversely, low levels of self-acceptance are associated with depression (53, 54), and symptom severity in major depressive disorder is negatively associated with being self-soothing when experiencing negative emotions (55) as well as accepting and tolerating negative emotions (56). Therefore, self-compassion may be a protective factor against depression.

These findings suggest that higher levels of self-compassion may protect against depression, and help depressed individuals cope with stress and adversity. Self-compassion may also enhance social functioning and well-being, as it may foster empathy, trust, and cooperation with others. Therefore, it is important to address the deficits in self-compassion in the treatment of depression and to improve the social outcomes of depressed individuals.

### 3.4 Low socio-cognitive flexibility

Cognitive flexibility is the ability to adjust one’s cognitive strategies or behaviors in response to environmental changes, allowing for greater adaptability and problem-solving skills in social interactions (57, 58). Research has shown that cognitive flexibility is negatively correlated with depressive symptoms and mediates the relationship between coping styles and resilience, highlighting its importance for mental health (59) (see **Figure 1**). Depressed individuals tend to exhibit rigid and ruminative thinking styles, making it difficult for them to adapt to changing situations (60, 61). Additionally, individuals with depression exhibit compromised ability to shift attention and response from one emotional category to another (62), as well as deficits in the ability to respond appropriately to changing environmental requirements (63). Self-report measures have also revealed that greater levels of depression are associated with lower scores of cognitive flexibility and higher levels of impulsivity (64). Furthermore, experimental studies have shown that MDD patients exhibit higher switch costs in reaction times, indicating decreased cognitive flexibility (65). Therefore, cognitive flexibility may be impaired in depression and affect various aspects of functioning.

These findings show that depression is associated with low cognitive flexibility and high cognitive rigidity which may affect their social functioning and lead to more social problems and conflicts. Therefore, it is important to address these impairments in the treatment of depression and to improve the social outcomes of depressed individuals.

### 3.5 Low capacity to forgive

Forgiveness is a multifaceted construct that involves considering relationships in a balanced manner, recognizing others’ weaknesses and mistakes, as well as their strengths and talents. The ability to forgive and repair relationships has been linked to a reduction in depression, indicating its effectiveness in regulating negative affect (66, 67). In fact, forgiving others leads to a decrease in depressive and angry emotions, thoughts, and behaviors, and an increase in positive and benevolent ones toward the offending person (68).

Studies across all ages have found that both trait and state forgiveness are significantly associated with lower rates of depression (69). Moreover, forgiving others has been inversely linked to depressive symptoms (70–72), including postpartum depression (73) (see **Figure 1**). Recent research has highlighted that participants who were more benevolent and forgiving demonstrated greater self-reassurance and reported lower depressive symptomatology (74). Conversely, a lack of forgiveness may be considered a predisposing factor to depression (75), since it perpetuates negative emotions related to perceived offences in relationships ( (76, 77). Thus, the evidence suggests that forgiveness plays a crucial role in regulating negative affect and mitigating the risk of depression across all ages.

These findings suggest that forgiveness can reduce depression and negative affect and increase positive and benevolent emotions toward others. However, a lack of forgiveness may be a risk factor for depression, as it perpetuates negative emotions related to perceived offences in relationships. Therefore, it is important to address the deficits in forgiveness in the treatment of depression and to improve the social outcomes of depressed individuals.

### 3.6 Deficits in emotion recognition and perception

Social functioning in depression may be impaired by a distorted perception of emotions and mental states. Depressed patients tend to interpret social stimuli in a negative way that matches their mood. For example, they show more attention to sad faces and less to happy faces (78). They also have a higher sensitivity to neutral faces that were previously associated with sad expressions, suggesting a negative memory bias (79). Moreover, depressed patients have difficulty recognizing subtle or complex emotions (80) (see **Figure 1**). It has been shown that dysphoric individuals tend to perceive ambiguous faces as negative (81). Therefore, the inability to regulate or correct the negative bias may increase the vulnerability to depression (82). These findings indicate that depressed patients have deficits in emotion recognition and a mood-congruent negative bias that affect their social functioning. They tend to perceive social stimuli in a more negative and less positive way than non-depressed individuals and have trouble identifying and interpreting complex or ambiguous emotions. These impairments may lead to reduced social engagement, increased interpersonal conflicts, and lower social support.

These findings suggest that depression impairs the perception of emotions and mental states in social situations leading to impairment of social functioning and leading to more social isolation and conflict. Therefore, it is important to address these deficits in the treatment of depression and to improve the social outcomes of depressed patients.

### 3.7 Deficits in empathy

Empathy is essential for understanding others by taking their perspective, managing emotions and navigating social situations (83). However, empathy seems to be compromised in depression. Depressed patients show less sensitivity to others’ pain (84), and lower cognitive empathy, as reflected by poor perspective taking, theory of mind, and empathic accuracy (85) (see **Figure 1**). On the other hand, higher affective empathy may increase the risk of depression, as it may lead to excessive guilt and responsibility for others’ suffering (86).

These findings suggest that empathy may have a dual role in depression: it may facilitate social functioning, but also increase the risk of depression. It is important to explore how empathy can be modulated and optimized in the treatment of depression and to improve the social outcomes of depressed patients.

### 3.8 Increased sensitivity to social rejection

Depression may make people more sensitive and avoidant of social signals and rejection (87). Depressed individuals may also expect and confirm negative social outcomes by ignoring positive information (88). As a result, they tend to perceive more social rejection than others (1, 89, 90) (see **Figure 1**). Moreover, they may find rejection more distressing (91) and react more negatively to it (92). Even after recovery, they may remain more vulnerable to interpersonal criticism (93). These impairments may impair social functioning in depression and increase isolation and loneliness. Therefore, it is important to address these impairments in the treatment of depression and to improve the social outcomes of depressed individuals.

### 3.9 Decreased social connectedness

Social connectedness is the feeling of closeness and belonging to other people and groups . People who have high social connectedness tend to enjoy social interactions, see others as friendly, and relate to them easily (94). However, depression is linked to low social connectedness and integration. Chronically depressed patients may feel less connected and compassionate to their close ones (see **Figure 1**). They may also experience more depression as a result of low social connectedness, especially in adolescence (95, 96). Thus, depressed individuals may feel less close and compassionate to their close ones and may have difficulty enjoying social interactions and relating to others. A vital part of treating depression is therefore to help them cope with these troubles and to enhance their social quality of life.

## 4 Impairments of social behavior in MDD

### 4.1 Increased impulsivity and anger

Depression may be associated with higher impulsivity, according to a recent systematic review of 21 studies (97). Notably, depressed individuals may react more impulsively to both positive and negative emotions, indicating a general over-responsiveness to emotions (98) (see **Figure 1**). This may impair their ability to cope with stress (35) and negatively affect their social relationships (99–101). This higher impulsivity and emotional over-responsiveness may affect social relationships resulting in more interpersonal problems and conflicts. Therefore, it is important to address these issues in the treatment of depression.

### 4.2 Decreased prosocial behavior

Depression may diminish the joy and motivation of helping others. Prosocial behavior, or voluntary actions that benefit others, has been found to be inversely related to depressive mood in non-clinical samples (102–104). Further, depressed individuals may be less inclined to help others because they expect less positive outcomes from social situations (104) (see **Figure 1**). This can lead to reduced social support and happiness. Thus, helping depressed patients to overcome these problems is a key part of treating depression and enhancing their social well-being.

### 4.3 Deficits in social decision making

Social behavior depends on the ability to evaluate the fairness of social interactions and to cooperate with others. Since depression may affect various aspects of social decision-making, such as fairness, cooperation, trust, and reciprocity, several paradigms have been used to study these abilities, such as the Ultimatum Game (UG), the Prisoner’s Dilemma (PD) and the Trust Game context. The UG involves two players, one of whom proposes how to split a sum of money and the other who accepts or rejects the offer. If the offer is rejected, neither player gets anything. Depressed patients tend to make more fair offers as proposers (105, 106) and to reject more unfair offers as responders than healthy controls (see **Figure 1**). They also reject more offers when they are paired with emotional facial expressions. Their rejection rate is correlated with their depression severity and does not change after treatment. Depressed individuals may also have a higher preference for fairness and a desire to punish unfair actions, which may be related to decreased forgiveness of others. This behavior of depressed patients may be influenced by several factors, such as fear of rejection , reduced reward sensitivity as well as negative emotions, pessimism, and negative associations (105, 107, 108).

The PD is a game where two players can choose to cooperate or defect, resulting in different payoffs depending on their choices. Cooperation is optimal for both players, but defection is tempting for each individual (109). Depressed patients tend to cooperate less than healthy controls (108, 110) and have more negative emotional reactions to betrayal . Their cooperation may depend on their partner’s behavior: they are more prosocial with cooperative partners but more volatile with unbiased partners (111).

The Trust Game is a game where one player can send some money to another player, who can then return some or none of it. The amount sent by the first player is multiplied by a factor before reaching the second player, creating an incentive for trust and reciprocity (109). Depressed patients show less reciprocity than healthy controls in the Trust Game, except when they are in remission. They may also be more sensitive to the risk of being cheated by their partner.

These findings show that depression affects the ability to evaluate the fairness of social interactions and to cooperate with others. The tendency to make fair offers and reject more unfair offers in the Ultimatum Game may reflect the fear of rejection, reduced reward sensitivity, and higher preference for fairness. They also tend to cooperate less and react more negatively to betrayal in the Prisoner’s Dilemma, which may reflect their pessimism, volatility, and negative emotions. Showing less reciprocity and more risk sensitivity in the Trust Game, which may reflect their distrust and low self-esteem. These impairments may affect their social functioning and lead to more social isolation and conflict. Therefore, it is important to address these impairments in the treatment of depression and to improve the social outcomes of depressed individuals.

## 5 Diagnosis of social deficits in MDD

Overall, our findings demonstrate that recovery from depression requires significant improvement not only of depressive symptoms but also in social adaptation and functioning. Assessing the severity of MDD only through disease-specific symptoms is therefore insufficient to understand a patient’s needs and the personal burden of disease. We therefore recommend that screening, treatment, and therapeutic approaches should not only target core depressive symptoms but also integrate an assessment of the patient’s social functioning and social network. These assessments could include both subjective measures of social dysfunction, such as the Social Functioning Questionnaire (112), Social Dysfunction Rating Scale (113) or Work and Social Assessment Scale (114), as well as objective measures, such as longitudinal changes in a patient’s social decision-making tendencies in neuroeconomic games (115). Such measures could be used as diagnostic markers for clinical diagnoses and measures of treatment outcomes.

Furthermore, social networks may provide opportunities to screen people for depression, as people are increasingly relying on social media to search for social connections, share emotions, and post about their daily activities. Depressed and non-depressed individuals differ in various measures of social media use, including the number and content of Facebook messages, linguistic variability in tweets, number of followers, and frequency of Instagram use (116). Having fewer Facebook friends or mutual friends, posting frequently, and describing negative emotions, as well as the frequent use of personal pronouns, words related to pain and aggression, or “past focus” words, have all been positively correlated with depression (116). Visual content on social media sites (e.g., photographs posted to Instagram) can also be used as a marker for detecting depression. For example, markers of depression can be observed in the behavior and posts of Instagram users even before the date of the first depression diagnosis (117). Given these social media “signatures” of depression, automated analysis of social media could potentially be used for early detection of depression. If an automated process could detect elevated depression scores in a social media user based on their online content, that individual could be targeted for a more thorough assessment and provided with resources, support, and treatment. Furthermore, automated detection methods that rely on artificial intelligence and machine learning for passive, large-scale monitoring of social media sites may help identify depressed or otherwise at-risk individuals (118–121). For example, the use of certain words (122), as well as other patterns in a user’s language and online activity, could indicate depression (118). Machine learning-based analyses of social media could therefore serve as an additional tool in public mental health screening (Liu et al., 2022), help physicians diagnose mental illnesses, and help psychiatrists analyze patient behavior (123).

## 6 Well-established treatments of social deficits in MDD

There are a wide variety of biological (i.e., pharmacotherapeutic) and psychological (i.e., psychotherapeutic) therapies available for social deficits in depression; however, these traditional models of treatment delivery have several limitations.

### 6.1 Pharmacotherapy

Antidepressants are the mainstay of pharmacological treatment for depression, but their effects on social functioning are limited and inconsistent. Antidepressants are primarily aimed at relieving the acute symptoms of depression and restoring the mood, rather than improving social functioning (124). Even when antidepressants reduce depressive symptoms, they often fail to restore normal levels of social functioning in various domains, such as social relationships, occupational and marital functioning, and social support life (125). Moreover, antidepressants may have a negative impact on some aspects of social functioning, such as empathy and emotional reactivity, by reducing the aversive responses to the suffering of others or inducing emotional blunting (126–128).

Among the novel antidepressants, only desvenlafaxine and vortioxetine have consistently improved social functioning, as measured by an improvement in mood and social relationships (129, 130). Some antidepressants, such as citalopram and reboxetine, have also shown some positive effects on social cognition tasks involving facial affect recognition (131, 132) and interpretation of affective pictures (133, 134) (see **Figure 1**). However, these effects are often small and do not match the much larger changes typically observed for classical symptoms of depression (135, 136). Moreover, these effects are not fully explained by changes in depressive symptom severity and may not translate into real-life social situations (137, 138). Thus, efficacy in relieving the core symptoms of depression does not necessarily guarantee efficacy in relieving impaired social functioning.

Furthermore, antidepressants may have a delayed effect on social functioning compared to mood symptoms. Two Japanese studies showed that antidepressants improved social adaptation after four weeks of treatment, whereas mood symptoms improved after two weeks (139, 140). This suggests that improvement in social functioning tends to lag considerably behind clinical remission from severe depression. Therefore, antidepressants may not be sufficient to address the complex social needs of individuals with depression.

### 6.2 Psychotherapy

Psychotherapy is another treatment option for depression that can improve social functioning by addressing the interpersonal and emotional aspects of the disorder. It can be combined with antidepressants to achieve better outcomes than medication alone (141).

Psychotherapy approaches can have varying effects on social functioning depending on their specific goals and focus. For instance, a systematic review of five studies aimed at addressing social isolation and depression in aged care clients (142) found that reminiscence therapy was the only approach that significantly reduced both social isolation and depression (143). In contrast, Interpersonal Psychotherapy (IPT) is specifically designed to enhance social functioning. IPT achieves this by helping patients manage new roles within their relationships and resolving interpersonal conflicts (144). IPT has demonstrated its effectiveness in several ways, including bolstering social skills, improving social support, fostering healthier parent-adolescent relationships, and enhancing attachment security (145).

Cognitive behavioral therapy (CBT), another influential psychotherapy, targets mood and cognition improvement. This is achieved by challenging negative thoughts and behaviors while imparting coping skills (146). Although a number of studies indicate that adding to pharmacotherapy produces only moderate improvement in social functioning (146–148), other studies have shown that it can reduce loneliness (149) and improve social functioning (150). Thus, several studies have indicated that CBT can alleviate loneliness and promote better social functioning (149, 150). Moreover, CBT can also be effectively employed to address issues like social media addiction, a problem that can negatively impact both social functioning and overall life satisfaction (151).

A different approach to enhancing social functioning is the Cognitive Behavioral Analysis System of Psychotherapy (CBASP). CBASP seeks to improve social functioning by facilitating corrective interpersonal experiences within the patient-therapist relationship and teaching effective problem-solving techniques (152). Research has shown that CBASP can lead to an increase in empathic behavior (see **Figure 1**) and a reduction in interpersonal problems among individuals dealing with depression (153).

In conclusion, psychotherapy is a valuable treatment option for depression that can improve some aspects of social functioning by addressing the interpersonal and emotional aspects of the disorder. However, although CBT and IPT might reduce social deficits (145, 154), they do not fully ameliorate impairments in social functioning (23). In addition, many patients do not accept behavioral treatments and they are associated with high drop-out rates and they may not prevent relapses (155). Therefore, it is important to explore additional treatment options that can target the underlying neurobiological and psychological mechanisms of social functioning in depression, such as novel pharmacological agents or psychosocial interventions.

### 6.3 Electroconvulsive therapy

Between 12% (156) and 30% (157) of individuals with depression do not respond to consecutive courses of antidepressant medication and psychosocial therapy, resulting in a diagnosis of treatment-resistant depression (TRD). For individuals with TRD, various somatic or brain stimulation therapies may be considered. Electroconvulsive therapy (ECT) has proven to be an effective option for TRD (158). However, ECT does not consistently affect negative neurocognitive bias related to social impairments in major depressive disorder (MDD), as measured by the amygdala response to emotional faces (159). Similarly, experimental animal studies utilizing electroconvulsive seizure did not reverse the negative effects of chronic social stress exposure in mice; instead, they provided insights into potential mechanisms contributing to ECT side-effects in humans, such as short and long-term memory loss (160). Nevertheless, a study has suggested that ECT’s effect on memory may have a beneficial effect related to social functioning by reducing negative memory bias (161).

## 7 Novel treatments of social deficits in MDD

### 7.1 Transcranial magnetic stimulation

Transcranial magnetic stimulation (TMS) is a non-invasive technique that modulates neural activity in the brain by generating a magnetic field through a brief discharge of electric current into a stimulated coil. This magnetic field induces depolarization of neural cell membranes in cortical tissue under the coil, affecting related nerve activity loops. It has shown the ability to improve social performance in depressed patients (162) by modifying cognitive control processes related to emotion regulation (163). Specifically, TMS targeting the right ventrolateral prefrontal cortex (PFC) has been shown to mitigate negative emotions experienced during social exclusion situations in depressed patients, suggesting its potential to enhance emotional regulation in response to social pain and social rejection (164).

### 7.2 Transcranial direct current stimulation

Another noninvasive neuromodulation technique, transcranial direct current stimulation (tDCS), has been employed successfully to induce mood changes in individuals with depression. Activation of the right ventrolateral PFC using tDCS has positively impacted emotional regulation (see **Figure 1**) while viewing images of social exclusion, particularly when reappraisal techniques are employed (165). Moreover, tDCS over the left dorsolateral PFC has reduced perceived emotional valence for negative images, potentially through enhanced cognitive control over emotional expression (166). Additionally, tDCS has improved interference control in tasks involving sad and neutral faces, as evidenced by faster reaction times (167). Furthermore, tDCS of the PFC has enhanced cognitive reappraisal of negative stimuli in depression (168), while also reducing aggressive responses to social exclusion (169). Moreover, tDCS may have a profound impact on compassion motivation, as revealed in a recent study employing anodal transcranial direct current stimulation (tDCS) on the right insula (170). Compassion motivation, linked to a calm physiological state indicated by heart rate variability (HRV), was significantly influenced by this tDCS technique. It increased HRV during videos evoking empathy, rendering individuals more sensitive to distress signals while concurrently reducing negative feelings and amplifying positive ones during subsequent tasks. These results suggest that tDCS may enhance compassion by modulating the physiological and emotional aspects of compassion motivation, potentially facilitating more empathetic and socially attuned behavior.

In summary, for individuals with treatment-resistant depression, therapies such as ECT, TMS, and tDCS hold promise in improving social functioning and social interactions. These therapies can influence emotional regulation, cognitive control, and memory processing, all of which are critical components of social behavior and social relationships in individuals with depression.

### 7.3 Vagus nerve stimulation

Vagus nerve stimulation (VNS) offers an alternative approach to treating depression by modulating various brain networks (171) and holds great potential for addressing not only mood-related symptoms but also social deficits commonly observed in depression. The vagus nerve may be an important mediator between depression and social functioning since it connects inner organs such as gut, heart and brain with social expressions, including pitch and tone of the voice as well as facial expression. Given that vagus nerve activity is reduced in MDD (172), therapeutic modulation of its activity has the potential to improve depressive symptoms and social functioning. Historically, VNS required surgical implantation, but a recent development called transcutaneous vagus nerve stimulation (tVNS) eliminates the need for surgical implants. Instead, it employs a bipolar electrode attached to the skin of the left ear conch (173).

In healthy subjects, tVNS has demonstrated promise in enhancing emotional processing and cognitive regulation and the sense of connection. It has improved cognitive reappraisal skills and reduced the perceived intensity of emotion-eliciting pictures (26). Additionally, tVNS has shown the ability to modulate attention to direct gaze (174) and enhance emotion recognition for faces (175). Significantly, transcutaneous electrical stimulation of the auricular branch of the vagus nerve (taVNS) has been found to selectively direct visual attention toward facial features essential for emotional recognition (176). Moreover, this stimulation has been associated with elevated levels of oxytocin in saliva, a neurotransmitter pivotal in modulating social cognition.

Furthermore, tVNS has been observed to enhance emotion recognition, especially for easily discernible emotional cues (177). Recent investigations have revealed that tVNS can reduce emotional biases toward both sad and happy facial expressions (178). In adolescents diagnosed with MDD, tVNS has been found to alter early visual processing of negatively valenced emotional stimuli, potentially reducing responses to sad emotional stimuli (179). These findings suggest that tVNS may reduce emotional reactivity, particularly toward sadness, and enhance the ability to decode social cues, potentially improving social functioning and facilitating more positive social interactions and relationships.

### 7.4 Intranasal oxytocin

There are also several novel treatments for depression that are less rooted in electrochemical physiology. In addition to using TMS and tVNS to treat social impairment, numerous studies over the last two decades have explored the modulatory role of the neuropeptide oxytocin on social cognition and behavior (180). In healthy participants, intranasal oxytocin administration led to increased eye contact with others, thus improving facial recognition (181) and body language-based emotion recognition (182). In persons at risk for depression, administration of oxytocin facilitated the processing of positive emotional cues (see **Figure 1**), enhancing the maintenance of attention to the mouth region of happy faces (183). Furthermore, intranasal oxytocin administration improved mood in new mothers who otherwise reported a moderately low mood (184). In mothers with postnatal depression, oxytocin can amplify responses to happy expressions from infants (185). In a related study, mothers with postnatal depression who received intranasal oxytocin were more likely to rate an infant cry as “urgent” (186). Another study has shown that after administration of oxytocin, postnatally depressed mothers were more protective of their baby in the presence of a stranger (187). For these reasons, oxytocin is worth investigating in terms of improving social functioning in MDD.

### 7.5 Intravenous and intranasal ketamine

Over the past two decades, subanesthetic ketamine treatment has emerged as a groundbreaking approach for addressing treatment-resistant depression (TRD) (188), offering rapid and immediate relief to patients who have previously found little success with other treatments (189). This development has instilled hope among individuals grappling with TRD, representing a promising alternative (158). Ketamine therapy encompasses a racemic mixture comprising two enantiomers, R-ketamine (arketamine) and S-ketamine (esketamine). The Food and Drug Administration (FDA) recognized the efficacy of intranasal esketamine, marketed as ‘Spravato,’ in conjunction with oral antidepressants for TRD treatment, thus offering a novel method of administration (190).

In contrast to traditional antidepressants, which typically require weeks to manifest therapeutic effects, both ketamine and serotonergic psychedelics have shown the capacity to alleviate depressive symptoms and enhance emotion regulation and social functioning (29, 158, 191, 192) within hours following a single administration (193, 194). For example, even a rapid intravenous ketamine injection at a dose of 0.5 mg/kg has proven effective in reducing rumination (195) and inducing sustained improvements in negative self-schema (196).

Ketamine’s positive impact on social functioning extends to its ability to enhance neural responses to positive emotions (see **Figure 1**), particularly within the right caudate, which appears to correlate with reduced depression severity (197). In the context of MDD, ketamine infusions have led to increased motivation and desire to engage in social interactions (158). Post-treatment, individuals with depression have reported a shift in their perceptions of others, reporting increased feelings of closeness, connectedness, and an enhanced ability to relate (198). These experiences are noteworthy as they suggest that ketamine can induce alterations in self-perception and reduce self-focus, potentially contributing to improved social interactions and emotional well-being. Additionally, research findings from another study indicate that ketamine has the capacity to mitigate pathological self-focus during emotional processing tasks. Specifically, it has been observed that ketamine administration results in increased deactivation of the Default Mode Network (DMN), particularly in response to negative and aversive stimuli, when compared to a placebo in healthy subjects assessed 24 hours after administration (199). This finding implies that ketamine may facilitate a more flexible and adaptive responsiveness of the DMN, reducing excessive self-referential processing during emotionally charged situations.

Recent research has also shed light on ketamine’s role in restoring top-down control of emotion processing. Thus, ketamine’s potential as an antidepressant extends to its ability to normalize brain function during emotionally valenced attentional processing (200). Additionally, by increasing functional connectivity between limbic regions and the central executive network, which plays a crucial role in top-down processing necessary for effective emotion regulation, ketamine may facilitate better emotional control (201). The rapid and positive effects of ketamine on emotional processing not only hold promise for improving social functioning but also have the potential to facilitate the establishment of therapeutic relationships, a cornerstone of various psychotherapies (191).

Ketamine has also been shown to reduce anhedonia, which could lead to improvements in social functioning (202). The research reported significant reductions in anhedonia following repeated ketamine infusions in patients with treatment-resistant depression. Notably, these effects were more pronounced in patients not using benzodiazepines, suggesting that ketamine’s impact on anhedonia might be influenced by concurrent medication use. However, larger placebo-controlled trials are needed to validate these findings conclusively.

The molecular mechanisms underlying the antidepressant actions of ketamine remain incompletely understood (203). At the neuronal level, esketamine holds the potential to bring about significant enhancements in neural plasticity and synaptogenesis, primarily through the upregulation of brain-derived neurotrophic factor (BDNF) production. Stress and depression often manifest as decreased BDNF levels in key brain regions, including the prefrontal cortex and hippocampus (30–32). Notably, studies have indicated that chronic ketamine treatment can effectively counteract the decline in BDNF protein levels in critical regions such as the hippocampus and nucleus accumbens in a rat model of depression (204). These findings suggest that ketamine-induced BDNF release may facilitate the adaptive rewiring of pathological neurocircuitry, ultimately leading to enduring structural brain adaptations that can sustain therapeutic benefits even in the absence of chronic dosing (205). Moreover, it has been postulated that the rapid antidepressant effects of esketamine may be closely linked to synaptic potentiation, a phenomenon that could contribute to a reduction in negative thinking patterns (206). By fostering synaptic potentiation, esketamine might facilitate the formation of new neural connections and the strengthening of existing ones, potentially improving emotional regulation and cognitive processes.

The pharmacodynamics of ketamine are intricately intertwined with multiple neurotransmitter systems, making its mechanisms of action complex. Notably, its rapid alleviation of depressive symptoms and reduction of suicidality have been attributed to its modulation of the NMDA receptor and interaction with the opioid system, as evidenced in a rat model (207). Furthermore, recent research has expanded our understanding of the endogenous mu-opioid system, revealing its involvement in more than just the hedonic aspects of pain. It has been proposed that the mu-opioid system plays a role in reinforcing socially affiliative or protective behavior in response to both positive and negative social experiences, with potential long-term implications for social behavior and health (208).

Furthermore, ketamine’s impact on the serotonin system extends beyond the traditionally recognized 5HT2A/1A receptors, known for their association with antidepressant effects. Ketamine may also activate other serotonin receptors, potentially influencing mood and social behavior. In rodents, ketamine’s antidepressant properties involve both AMPA and 5-HT1B receptors, with low 5-HT1B receptor binding observed in limbic regions in Major Depressive Disorder (MDD). However, human studies have shown that ketamine treatment does not significantly alter 5-HT1B receptor binding overall, although an increase in binding is noted in the hippocampus, suggesting a potential role of 5-HT1B receptors in ketamine’s antidepressant action (209). Additionally, considering the significant role of the glutamatergic system in MDD and social functioning, the surge in glutamatergic activity following ketamine administration is believed to contribute to its rapid antidepressant effects (210).

The observed efficacy of ketamine in depression management underscores its potential utility, particularly in cases where conventional treatments prove ineffective (211). For a substantial subset of depressed individuals facing challenges with traditional therapies, ketamine’s swift antidepressant actions hold promise for enhancing overall well-being and fostering a sense of connectedness and existential fulfillment (212).

### 7.6 Novel psychotherapeutic interventions

In response to the limitations of conventional treatments for social impairments, several researchers have investigated the effectiveness of novel psychotherapeutic interventions. Two such examples include the “Cognitively Based Compassion Training Programme” for couples (CBCT^®^-fC), which aims to improve social interaction skills (213), and the Cognitive and Emotional Recovery Training Program for Depression (CERT-D), which aims to improve psychosocial functioning (214). Additionally, various other psychotherapeutic methods hold promise in improving social functioning for individuals experiencing depression. These approaches include social competence training, emotion regulation skills training, and emotional intelligence training. Each of these methods focuses on specific aspects of social functioning such as social competence training (215), emotion regulation skills training (216, 217), or emotional intelligence training (218).

### 7.7 Mindfulness-based cognitive therapy

Emerging as promising novel treatment approaches deeply rooted in the spiritual traditions of indigenous and religious communities, both mindfulness-based cognitive therapy (MBCT) and psychedelic-assisted psychotherapy (PAP) have garnered significant scientific attention (219, 220). These therapeutic modalities, originating from diverse cultural contexts, have recently demonstrated the potential for complementary effects on enhancing social functioning in individuals grappling with depression (221).

In the realm of MBCT, a structured program integrates mindfulness-based training interventions with elements of cognitive behavioral therapy (222). These interventions are meticulously designed to actively manipulate attention and awareness. Notably, they have proven effective in diminishing self-centered thought patterns (223, 224) and reducing emotional reactivity to the rigors of social stress (225).

One of the core mechanisms through which MBCT contributes to improved social functioning in individuals with depression is the cultivation of self-awareness and self-regulation. By actively altering attention and awareness, MBCT enables individuals to gain insight into their thought processes, emotional reactions, and habitual patterns of behavior. Thus, a recent study has shown that MBCT might effectively reduce negative self-bias and facilitate psychophysiological benefits associated with a more positive self-view (226).

Another mechanism by which mindfulness enhances social functioning in individuals grappling with depression is cognitive reappraisal. Evidence suggests a strong correlation between mindfulness training and the augmentation of cognitive reappraisal as an effective emotion regulation strategy. For instance, one study revealed that individuals who received brief mindfulness training exhibited substantial increases in dispositional nonreactivity, a fundamental aspect of mindfulness (227). Importantly, this heightened dispositional nonreactivity was positively linked with the increased adoption of cognitive reappraisal techniques over time.

Mindful decentering, a core element of mindfulness, plays a pivotal role in facilitating positive reappraisal (228). It involves disengaging from initial appraisals of events and entering a state of metacognitive awareness that reduces semantic evaluations associated with those events. This cognitive shift, inherent to mindfulness, allows individuals to attribute new, more positive meanings to previously distressing situations. By cultivating mindfulness consciousness, individuals can redefine and reframe their circumstances in a way that fosters hope and resilience - a crucial component of meaning-based coping strategies.

In essence, mindfulness, with its capacity for cognitive reappraisal, becomes an integral tool for individuals facing acute or chronic stressors. The natural state of mindfulness, which involves cognitive flexibility, enables individuals to make positive reappraisals even in challenging social situations. Therefore, mindfulness training has the potential to empower individuals to effectively manage their emotions by encouraging them to reevaluate and reinterpret their emotional experiences. This, in turn, contributes significantly to their emotional well-being and improved social functioning.

Moreover, mindfulness has been associated with heightened compassion, extending both to oneself (self-compassion) and others (229). The heightened compassion toward others fosters a profound sense of interconnectedness and facilitates responding to potential conflicts with non-aggressive approaches (230). When individuals develop self-compassion, they are better equipped to manage self-criticism and self-blame, which are often intertwined with social difficulties in depression (231). This, in turn, can lead to more positive and constructive social interactions.

Recent research findings underscore the capacity of MBCT to effectively attenuate negative self-bias, thereby facilitating psychophysiological benefits associated with a more positive self-concept. This shift toward a more positive self-view can directly impact how individuals perceive their social interactions. As they develop a more positive self-image, they may feel more confident and less anxious in social situations, ultimately leading to improved social functioning (226).

Additionally, MBCT demonstrates a positive effect in diminishing self-criticism (232), curtailing ruminative thought processes (233, 234), mitigating self-blame (231), and reducing shame-proneness (235). These changes in cognitive and emotional processes may contribute to more adaptive social behaviors and interactions, as individuals are less burdened by negative self-perceptions and rumination, which can hinder social engagement.

Mindfulness practices offer valuable tools in addressing impulsive behaviors and anger among students. Thus, an older study has shown that mindfulness training can effectively decrease impulsive and aggressive behaviors (236). By cultivating present-moment awareness, mindfulness reduces obsessive rumination and promotes the experience of positive emotions by lowering the likelihood of engaging in impulsive behaviors, which often exacerbate the very emotional issues individuals seek to alleviate or resolve through aggression (237). Mindfulness training further equips individuals with the ability to recognize the initial signs of aggressive impulses, empowering them to inhibit these impulses effectively using the skills honed through mindfulness practice, such as acceptance and equanimity (236). In essence, mindfulness provides essential tools for reducing impulsivity and managing anger, promoting healthier responses to challenging emotions and situations.

Equally compelling is the evidence suggesting that MBCT augments cognitive flexibility (238–240). This can be highly beneficial in social interactions as it allows people to better understand diverse viewpoints and respond more skillfully to differing opinions and behaviors. In conflicts, cognitive flexibility can facilitate compromise and creative problem-solving, leading to healthier relationships.

In addition to enhancing cognitive flexibility, mindfulness practice also heightens empathetic capacities (83, 241). When individuals are more in touch with their own emotions and thoughts, they become more attuned to the feelings and needs of others. This heightened empathy can lead to improved communication, deeper connections, and a greater ability to provide emotional support in relationships.

Additionally, mindfulness training fosters compassion, and facilitates forgiveness toward others (230, 242). When individuals are more compassionate, they tend to be more patient and forgiving, which can lead to more harmonious and fulfilling relationships. Since holding onto grudges or resentment can strain relationships, being capable to let go of past grievances and focus on the present moment might facilitate healing leading to the restoration or improvement of relationships.

Finally, mindfulness training has been shown to increase willingness to cooperate in the ultimatum game (243). Further, two studies have shown that an 8-week mindfulness training program has promoted reactive prosocial behavior while observing suffering or hardship of a confederate (230, 244). Further, mindfulness promoted prosocial responsiveness to a representation of an ostracized stranger (245) (see **Figure 1**). Another study found that subjects in the mindfulness condition allocated more money to another participant despite not having any information about the financial needs or any other form of distress (246). When individuals are more mindful, they are more likely to respond to the needs of others in a helpful and altruistic manner. This can strengthen bonds, build trust, and contribute to a more positive social environment. The cooperative mindset can lead to better teamwork in various aspects of life, including personal relationships.

Notwithstanding these substantial advancements in social functioning attributed to MBCT, it is important to acknowledge that realizing these benefits often necessitates intensive and prolonged mindfulness practice. This acknowledgment underscores both the potential and the challenges of MBCT as an intervention in ameliorating social deficits among individuals contending with depression. Consequently, it highlights the need for further research to optimize the accessibility and efficiency of MBCT, ultimately ensuring its broad applicability in clinical settings.

### 7.8 Psychedelic-assisted psychotherapy

Psychedelic-assisted psychotherapy (PAP) has emerged as a therapeutic approach demonstrating analogous outcomes to MBCT, particularly in addressing social impairments associated with depression. PAP encompasses the administration of psychedelic substances, including MDMA, psilocybin, LSD, ayahuasca, ketamine, or ibogaine, within a structured intervention coupled with psychotherapy (247, 248). Within this structured context, psychedelic agents have exhibited the capacity to induce enduring positive alterations in social functioning following a single dosage (249). Thus, a single ingestion of ayahuasca in a social setting resulted in increased cognitive empathy as well as an improved ability to recognize emotions in others when compared to baseline (250) (see **Figure 1**).

PAP facilitates the emergence of emotions like self-compassion, forgiveness, and self-acceptance, which serve as potent remedies for emotions such as shame, guilt, anger, isolation, disconnection, or other negative feelings that patients may struggle to address in therapy and that traditional antidepressants do not appear to alleviate (251).

One plausible mechanism underlying the improvement of social impairments in depression through psychedelics lies in their ability to modulate self-referential processes. Psychedelics have been found to diminish self-referential awareness (252), and ruminative self-focus (Letheby and Gerrans, 2017), facilitating a shift away from internalized negative thought patterns. This reduction in self-focused rumination can contribute to feelings of unity with others and an augmented sense of social affiliation (253, 254). By disrupting the dominance of depressive self-preoccupation, psychedelics may enable individuals to engage more fully in social interactions and connect with others on a deeper level.

Another mechanism might lie in the increased capability to adopt a decentered stance of mind after the use of psychedelics. It has been shown, that a single dose of ayahuasca led to increases in decentering ratings 24 hours and 7 days following the ingestion (250). These findings align with prior research that has consistently reported enhancements in decentering abilities and related mindfulness capacities at various post-ayahuasca time points, including 24 hours (255, 256), 15 days (257), and even 2 months post-intake (258). Previous research has shown that when individuals are prompted to analyze their feelings about negative autobiographical experiences from a self-distanced, decentered perspective (i.e., by visualizing events from the standpoint of an impartial observer) as opposed to a self-immersed, first-person viewpoint, they exhibit lower levels of acute emotional and physiological reactivity and are less prone to rumination over time (259, 260). This is highly relevant for depressive patients who often experience heightened emotional reactivity to social situations, which can manifest as increased sensitivity to perceived social rejection, criticism, or negative feedback and lead to acute emotional distress and physiological responses such as anxiety or even withdrawal from social interactions. By viewing negative social experiences as an impartial observer, depressed individuals might be able to reduce the intensity of their emotional reactions and decrease physiological arousal in social situations. Consequently, they may become less vulnerable to the negative emotional impact of social interactions and be more willing to initiate them.

Furthermore, psychedelics have been associated with the modulation of emotional processing. They have been found to ameliorate the negative emotional bias observed in depressed individuals during emotion recognition tasks. For instance, multiple studies have indicated that MDMA, commonly known as “ecstasy,” positively skews an individual’s interpretation of emotional facial expressions by augmenting the recognition of positive expressions while potentially impeding the recognition of negative expressions (261–264). This alteration in emotional processing may help individuals with depression perceive social interactions and cues in a more positive and socially attuned manner, ultimately improving their social functioning.

Numerous investigations have corroborated the enhancement of behaviors associated with cognitive flexibility and receptivity to novel experiences following psychedelic use (255, 265–271). This heightened cognitive flexibility may play a pivotal role in improving social impairments by enabling individuals to adapt more effectively to social contexts, read social cues, and engage in more fluid and adaptive social interactions.

Additionally, psychedelics have been associated with heightened emotional empathy (268, 272–275), prosocial behavior (249, 276), increased generosity (277), and self-reported altruism (278). These pro-social effects suggest that psychedelics may enhance individuals’ ability to connect with others on an emotional level, thereby addressing some of the social impairments often seen in depression (279).

Furthermore, psychedelics often induce a state known as ego dissolution, where the boundaries between self and others blur (280). This experience, commonly reported during psychedelic journeys (280, 281), suggests that psychedelics might promote a heightened sense of connection with others and potentially reduce feelings of loneliness. A more recent investigation focusing on male long-term AIDS survivors has suggested that psilocybin-assisted psychotherapy may effectively reduce attachment anxiety and enhance attachment security (282). Consequently, the effects of psychedelics seem to extend beyond individual well-being, offering the potential for therapeutic benefits in terms of improved social connections, better relationships, and more positive social behavior.

Furthermore, individuals consuming psychedelic substances have demonstrated a decreased likelihood of rejecting unfair offers in the ultimatum game (283) and a heightened inclination toward cooperative behavior in the prisoner’s dilemma (284). These findings suggest that psychedelics may promote more cooperative and adaptive social behaviors, potentially mitigating social difficulties commonly associated with depression.

Recent research highlights the potential of psychedelics, such as MDMA and psilocybin, to mitigate the impact of social rejection sensitivity, a phenomenon often exacerbated in individuals with depression. MDMA, for instance, has demonstrated a capacity to diminish the effects of simulated social rejection, as evidenced by improvements in self-reported mood and self-esteem, along with a reduced perception of rejection intensity within the context of virtual social interaction paradigm (Cyberball game) (285). Furthermore, an intriguing observation emerged from this research, indicating that higher doses of MDMA were associated with an increased estimated number of throws received by participants during the rejection condition in the Cyberball game. This suggests that MDMA may not only influence mood but could potentially alter the objectively assessed level of rejection experienced by individuals.

Similarly, psilocybin, administered to healthy volunteers, has exhibited the ability to reduce feelings of social exclusion (286). Intriguingly, during a Cyberball game, psilocybin was found to modulate emotional responses to social exclusion by attenuating activation in brain regions associated with social rejection (287). This dampening of social rejection sensitivity is of particular significance within the realm of psychotherapy. By ameliorating the emotional repercussions of perceived rejection, psychedelics like MDMA may create an environment conducive to open and honest communication. This effect holds significant therapeutic potential, offering individuals with depression a platform to engage more constructively with their emotions and interpersonal challenges during psychotherapeutic interventions.

Finally, the concept of connectedness was emphasized in psychedelic therapy, as revealed in a qualitative research paper for treatment-resistant depression (TRD) clinical trial (254). In 6-month follow-up interviews, participants, who endorsed the treatment’s effectiveness, consistently cited a key factor: a renewed sense of connection. This factor was categorized into three aspects: connection to oneself, others, and the world at large (254).

Psychedelics can potentially improve social functioning through their multifaceted effects on the brain and psychology. Their serotonin (5HT)2A agonist or partial agonist effects, have demonstrated rapid antidepressant properties, and this may contribute to their positive impact on social interactions. The modulation of the serotonin system can influence mood, emotional regulation and reward processing, potentially reducing symptoms of social anhedonia and social anxiety that hinder social interactions. Moreover, psychedelics have been shown to increase neuroplasticity, including effects on spinogenesis, synaptic pruning and brain-derived neurotrophic factor. Likewise, both healthy individuals and individuals with TRD exhibit heightened circulating levels of BDNF following a single administration of ayahuasca (288). Furthermore, psilocybin has been observed to elevate peak BDNF levels, irrespective of whether patients have received pre-treatment with the antidepressant escitalopram or a placebo, indicating that psilocybin exerts a significant influence on BDNF (289). These mechanisms are similar to effect of stimulating the vagus nerve in rats, which quickly increased the levels of neurotransmitters (such as norepinephrine) and the genes that produce neurotrophic factors like BDNF (290). These processes may help improve mood by promoting the growth of new brain cells in areas like the hippocampus, which are linked to mood disorders (291). Further, they could contribute to increased resilience to stressors in social settings (292).

From a neuroscientific perspective, studies using neuroimaging and neurophysiology have suggested that psychedelics may induce changes in brain connectivity. Specifically, they may disrupt the default mode network, consisting of the medial prefrontal cortex, posterior cingulate cortex, precuneus, and angular gyrus, which is typically overactive in conditions like MDD (293). Among individuals with depression, there have been documented decreases in functional connectivity within the DMN following psilocybin treatment (280, 294). More specifically, these reductions were noted in connections between the ventromedial prefrontal cortex (vmPFC) and the right angular gyrus (294). By altering the DMN, psychedelics may reduce self-referential thinking and introspection, potentially leading to more open and empathetic social interactions.

In summary, psychedelics have the potential to improve social functioning by acting on various levels. They may alleviate symptoms of depression and anxiety, enhance resilience to stress, and modify brain connectivity patterns, all of which can contribute to more positive and fulfilling social interactions. However, although clinical trials consistently showcase the efficacy of PAP across diverse mental health conditions, it is imperative to acknowledge that this area of research is still in its formative stages. It is important to note that further research is needed to fully understand the extent and nuances of these effects and to ensure their safe and responsible use. To comprehensively elucidate the potential moderators and mediators of this treatment modality and ascertain the sustainability of its effects over time, larger trials involving more heterogeneous participant samples remain a critical area of future exploration.

## 8 Role of animal models in treatment of social deficits

Animal studies using genetic, epigenetic, and environmentally-induced models of depression can help researchers test existing MDD treatments and search for novel therapeutics (295). Although human experiences of depression, such as sadness, guilt, or suicidal thoughts, cannot be simulated or assessed in rodents or other animal models, animals can still exhibit depression-related behaviors such as social anhedonia, social defeat, sociability, social anxiety, and social withdrawal (296). Animal models may therefore serve as a powerful tool for the study of social impairments, social behavior, and treatment options in depression (297). For example, raising rodents in constant social isolation immediately after weaning leads to behaviors typical of depression (298), such as despair-like behavior, which is expressed in mice as increasing immobility over time (299). The “social defeat stress” animal model (297) has been used to show that stress-susceptible mice exhibit repeated aggression and develop a long-lasting aversion to social contact. These behavioral changes can be reversed by administering antidepressants (300) or psychedelic drugs such as LSD or ketamine (301–303). For example, low doses of LSD increased sociability in mice by activating serotonin 5-HT2A receptors and alpha-amino-3-hydroxy-5-methyl-4-isoxazolepropionic acid (AMPA) receptors (304). Further, recent study in the rat model of depression has shown that after administration of ketamine, the rapid response entailed topological modifications in cognitive, sensory, emotion, and reward-related circuitry, whereas after 48 h ketamine had mainly mediator effects on cognitive flexibility (305).

Despite their evident imperfections, animal models could also provide invaluable help to understand the cellular and molecular mechanisms underlying the treatment of MDD (295). For example, Gerhard et al. (2020) recently demonstrated that in a mouse model, a specific NMDA receptor subunit on GABAergic interneurons was the initial cellular trigger for ketamine’s rapid antidepressant activity. Further, another study in mice has shown that in the chronic stress model, ketamine selectively reversed stress-induced branch-specific elimination of postsynaptic dendritic spines and led to remission of specific depression-related behaviors such as motivated escape behavior (306). This study has also shown that eliminating newly formed synapses abolished some of the drug’s positive effects, indicating that spinogenesis induced by ketamine might be useful for maintaining remission after ketamine treatment.

The use of animal models in psychiatric research, particularly in studies involving depression, raises significant ethical issues. Inducing depression-like states in animals often involves procedures such as chronic stress exposure, social isolation, and genetic manipulation, which can cause considerable distress and suffering to the animals (307). These methods, while valuable for understanding the mechanisms underlying depression and testing potential treatments, pose ethical dilemmas regarding the humane treatment of research animals.

To address these ethical concerns, several measures are implemented to ensure the welfare of animals used in research. All experimental protocols must undergo rigorous review and approval by Institutional Animal Care and Use Committees (IACUCs) or equivalent ethical review boards. These committees evaluate the scientific merit of the proposed studies and the ethical justification for using animals, ensuring that the research adheres to the principles of the 3Rs: Replacement, Reduction, and Refinement (308).

- Replacement: Whenever possible, alternative methods that do not involve animals, such as *in vitro* studies or computer simulations, are used to achieve the research objectives.
- Reduction: The number of animals used in experiments is minimized by employing efficient study designs and statistical methods that maximize the data obtained from each animal.
- Refinement: The procedures are refined to minimize pain and distress, including the use of analgesics, anesthetics, and humane endpoints to alleviate suffering.

Despite these measures, the ethical justification for animal research in psychiatry remains a topic of ongoing debate. Increasing community expectations regarding animal rights and welfare necessitate a continuous reassessment of the ethical frameworks guiding such research. Researchers are encouraged to maintain transparency in their methodologies and to engage in open dialogue with the public and ethical bodies to align scientific pursuits with evolving ethical standards (309).

## 9 Limitations

While this article embraces a narrative review approach, which provides a comprehensive overview of the literature, it is important to acknowledge some inherent limitations. Firstly, the subjectivity in selecting and interpreting studies could introduce bias into the review process. Additionally, the absence of a systematic search strategy might restrict the inclusion of relevant research.

Another limitation of this review is the lack of extensive exploration into the complexities of differentiating social deficits attributable to MDD from those arising from underlying personality traits independent of depressive episodes. Future research endeavors should aim to elucidate these distinctions, as they hold significant implications for accurate diagnosis and tailored treatment approaches. Despite these limitations, our review offers valuable insights into impairments of social functioning in depression and their treatments and serves as a catalyst for generating hypotheses for future research.

## 10 Summary and conclusion

In this comprehensive exploration of treatments for social deficits in MDD, we have reviewed a range of therapeutic approaches, encompassing both traditional and novel methods. While MDD is commonly associated with emotional distress and cognitive symptoms, its profound impact on social functioning is often overlooked. Traditional pharmacotherapy, represented by antidepressants, while effective in alleviating mood-related symptoms, has shown limited and inconsistent success in improving social deficits. Additionally, some antidepressants may even negatively impact certain aspects of social functioning. The integration of psychotherapy alongside pharmacotherapy has emerged as a promising strategy to enhance social functioning. Cognitive Behavioral Therapy and Interpersonal Therapy are two influential psychotherapeutic approaches that have demonstrated some success in mitigating social deficits associated with MDD. While these therapies can produce moderate improvements, they often fall short of fully ameliorating social impairments that importantly contribute to therapy discontinuation and depressive relapse. Therefore, it becomes imperative to explore additional avenues beyond these traditional approaches.

Brain stimulation therapies, such as electroconvulsive therapy, transcranial magnetic stimulation, and transcranial direct current stimulation, offer alternative paths for addressing social deficits in MDD. These therapies have shown promise in improving social performance, emotion regulation, and cognitive control. The diverse range of brain stimulation techniques suggests that modulating neural circuits holds significant potential for enhancing social functioning in individuals with depression, although further research is required to comprehend the underlying mechanisms fully.

Vagus nerve stimulation, especially through transcutaneous methods, introduces another non-invasive approach to treating depression’s social and emotional impairments. Emerging evidence suggests that transcutaneous vagus nerve stimulation (tVNS) can modulate emotional processing, attention, and social cognition. Its capacity to affect oxytocin release, a critical neuropeptide for social bonding, makes tVNS a compelling avenue for research and potential intervention in social deficits. In line with it, intranasal oxytocin administration offers a more direct pharmacological approach to enhance social cognition and behavior in individuals with MDD. Oxytocin has exhibited promising results in improving emotional processing, enhancing social interactions, and boosting mood in various contexts. Its potential to bridge the gap in social functioning is an area that might benefit from further exploration.

The emergence of ketamine treatment has marked a groundbreaking development in the field of depression therapy. Ketamine, particularly its S-ketamine enantiomer, has demonstrated rapid and substantial antidepressant effects, leading to its approval for the treatment of treatment-resistant depression. Ketamine’s impact extends beyond mood improvement, addressing emotional processing, social anxiety, and social functioning. Its ability to induce rapid and profound changes in emotional and social aspects makes it a promising intervention for social deficits in MDD.

Finally, mindfulness-based cognitive therapy (MBCT) and psychedelic-assisted psychotherapy (PAP) offer novel and complementary approaches to enhance social functioning in depression. MBCT combines mindfulness training with cognitive behavioral therapy, targeting self-focus, emotional reactivity, and self-criticism. In contrast, PAP utilizes psychedelic substances in conjunction with psychotherapy to induce lasting changes in social behavior, emotional processing, and cognitive flexibility. These two approaches, while distinct, share the common goal of improving social deficits, albeit with different mechanisms and timelines.

To gain a deeper understanding of the complex social deficits in MDD and the potential interventions, animal models have proven invaluable. These models allow researchers to explore treatment mechanisms at the cellular and molecular levels. Animal studies have uncovered insights into the action of antidepressants, psychedelics, and other therapeutic modalities, shedding light on the neural underpinnings of social deficits and their amelioration.

In conclusion, MDD poses significant challenges beyond the core emotional and cognitive symptoms, significantly impacting social functioning. Addressing these deficits necessitates a multifaceted approach that combines traditional pharmacotherapy and psychotherapy with innovative treatments like brain stimulation, vagus nerve stimulation, oxytocin administration, ketamine treatment, mindfulness-based cognitive therapy, and psychedelic-assisted psychotherapy. The integration of these diverse approaches offers hope for individuals with depression to regain a fulfilling social life, making it essential to continue researching and refining these interventions. By embracing this holistic perspective and advancing our understanding, we can better support those affected by depression in their journey toward improved social functioning and overall well-being.
